# Atypical presentation of myoepithelial hamartoma in the antrum of the stomach, mimicking a gastrointestinal stromal tumor: a case report

**DOI:** 10.1186/1752-1947-6-382

**Published:** 2012-11-12

**Authors:** Junaid Nabi, Fatema N Authoy, Sarker Mohammad Quamrul Akhter

**Affiliations:** 1 Department of General Surgery, Shaheed Suhrawardy Medical College and Hospital, Sher-e-Bangla Nagar, Dhaka, 1002, Bangladesh

## Abstract

**Introduction:**

A myoepithelial hamartoma is a very uncommon submucosal tumor of the stomach. In an atypical presentation in our case, it mimicked the clinical presentation of a gastrointestinal stromal tumor. To the best of our knowledge, it is the first case of a hamartoma of the stomach reported from Bangladesh and one of few cases described in the literature.

**Case presentation:**

We describe the case of a 35-year-old Bengali man with recurrent epigastric pain and occasional vomiting with radiographic findings of a gut mass. An upper gastrointestinal endoscopy revealed a healed duodenal ulcer, deformed ‘D’ bulb and a submucosal swelling in his antrum. Ultrasonography and a contrast-enhanced computed tomography scan confirmed the presence of a well-defined, oval gut mass in his upper abdomen, compressing his duodenum. The mass had a mixed density and was considered to probably be a gastrointestinal stromal tumor. Ultrasonography-guided fine needle aspiration cytology was inconclusive. After resection at laparotomy, a histopathological examination revealed a myoepithelial hamartoma. These tumors are characterized by hypertrophic smooth muscle bands surrounding varied epithelial elements, which may be arranged in diverse patterns such as simple glandular structure, Brunner’s gland, pancreatic ducts and sometimes pancreatic acini. This case report is complemented by a literature review relating to the atypical presentation.

**Conclusion:**

Gut masses need to be investigated thoroughly and the possibility of rare tumors should not be excluded. Although the recommended treatment for such lesions is limited resection, radical procedures such as a pancreaticoduodenectomy are often performed when the lesion occurs in the periampullary area because of preoperative misdiagnosis as a carcinoma. Therefore, it is essential for clinicians to maintain current knowledge of the lesion to avoid inaccurate diagnosis and prevent unnecessary surgery.

## Introduction

Submucosal tumors are extremely rare in the stomach, except for leiomyomas. Hamartomas are relatively common in the gastrointestinal tract; the preponderant tissues they contain are connective tissue derivatives including lamina propria, smooth muscle, vasoformative tissue, and nerve elements, though epithelial tissues also occur [1]. A myoepithelial hamartoma of the stomach, however, is a very rare submucosal tumor. Rare cases of ectopic pancreas and even fewer cases of hamartomas have been reported in the literature. In 1903, Magnus-Alsleben described for the first time the autopsy findings of hamartomas in five patients [2]; more recently, Vandelli *et al*. in 1993 published a review of the literature, still comprising only 33 cases [3,4]. According to indexed journals, no case of a hamartoma of the stomach has been reported in Bangladesh so far. Preoperative diagnosis is difficult for these cases because of the nonspecific symptoms as well as few cases being reported in the literature.

Here, we report a case of a myoepithelial hamartoma of the stomach where gastric endoscopy had revealed a healed duodenal ulcer and a submucosal swelling in the antrum, and a contrast-enhanced computed tomography (CT) scan had shown the presence of a gut mass that resembled a gastrointestinal stromal tumor (GIST).

## Case presentation

A 35-year-old Bengali man presented with complaints of intermittent pain in his epigastric region with occasional vomiting for two years. Over the last year, our patient had several episodes of heartburn followed by vomiting. The episodes lasted for a couple of days and occurred at an interval of two to three months. He denied any other symptoms. He was previously prescribed anti-ulcerant medication, which relieved him of his symptoms for a brief period of time. A physical examination was unremarkable.

**Figure 1:**
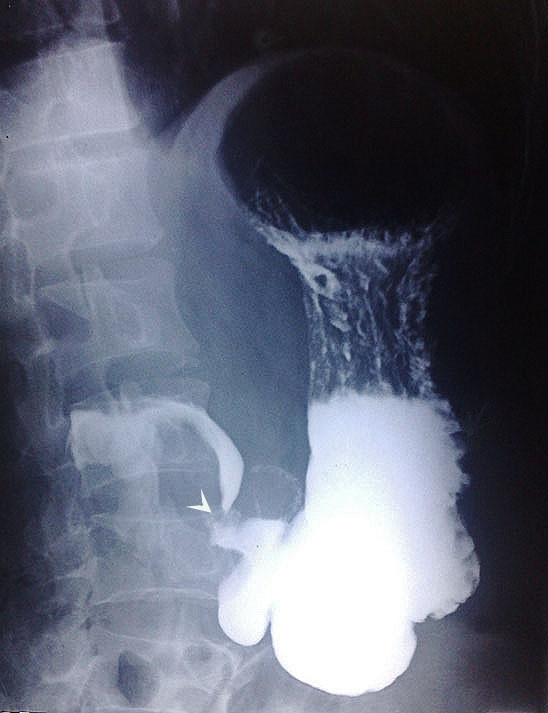
Barium meal follow-through showing a smooth filling defect, deformed bulb and partial narrowing of the duodenum (arrowhead).

**Figure 2:**
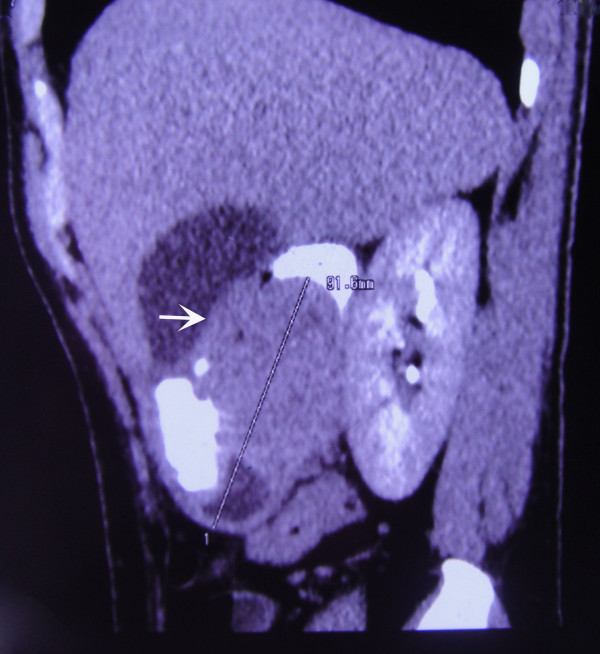
**Contrast-enhanced computed tomography of the gut mass (arrow).** Coronal section shows a well-defined, oval, soft tissue mass measuring 9.1×5×5.1cm in the upper abdomen, compressing the duodenum. Because of its location and nature, the mass was thought to be a gastrointestinal stromal tumor.

**Figure 3:**
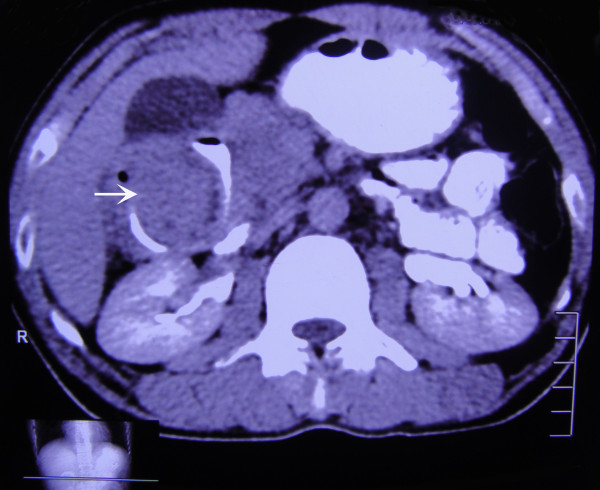
Contrast-enhanced computed tomography of the gut mass (arrow) in axial section.

In view of his history and clinical presentation, our patient was referred for an upper gastrointestinal endoscopy with the clinical diagnosis of peptic ulcer disease. Upper gastrointestinal endoscopy revealed duodenal ulcer disease in remission and a deformed bulb of the duodenum. An endoscopy 14 months before admission had shown erosions and a submucosal swelling at the antrum and an ulcer at the bulb of the duodenum, and an endoscopy made eight months before admission had shown a healed duodenal ulcer and partial narrowing of the channel. In view of his repeated intermittent abdominal pain, a barium meal follow-through of the stomach and duodenum was carried out. This showed the bulb of the duodenum to be deformed, with a smooth filling defect with eccentric linear passage of contrast in part of the duodenum (Figure 1) suggesting a diagnosis of a mass lesion, probably a GIST. On ultrasonography, a sharp-margined complex mass with both solid and cystic component was seen in his epigastric region superimposing on the first part of his duodenum. A contrast-enhanced CT scan confirmed the ultrasonography finding, showing a well-defined, oval, mixed density, soft tissue mass measuring 9.1×5×5.1cm in his upper abdomen, compressing his duodenum (Figures 2 and 3). The mass was separated from his pancreas, right kidney, gall bladder and liver. Ultrasonography-guided fine needle aspiration cytology showed blood products and a small number of mononuclear cells without anaplastic cells.

A diagnosis of GIST in the first part of duodenum was made.

**Figure 4:**
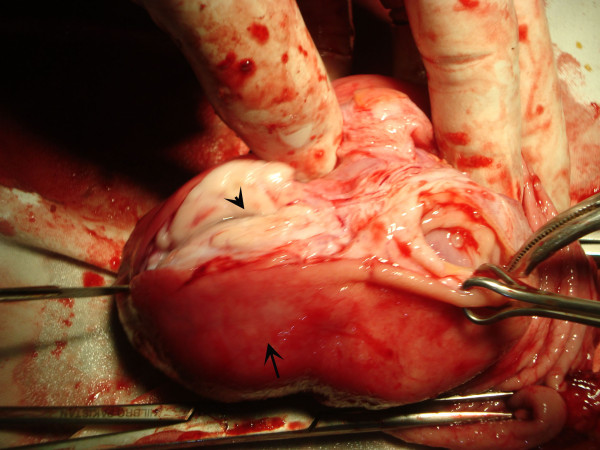
Perioperative macroscopic examination revealed a lesion (arrowhead) in the antrum of the stomach (arrow) extending 8cm along its longitudinal axis.

**Figure 5:**
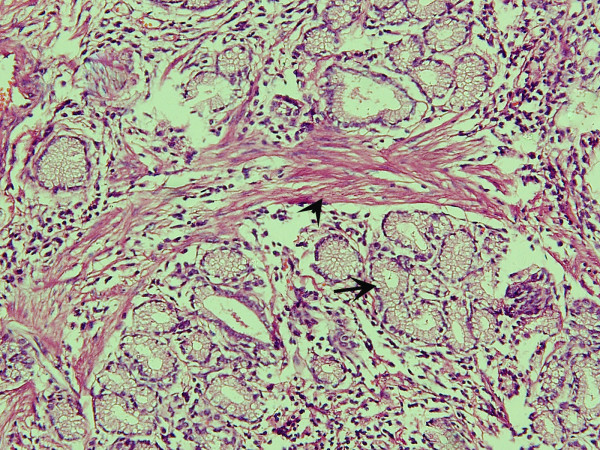
On microscopic examination, the lesion was composed of dilated pancreatic-type ducts (arrow) surrounded by smooth muscle bands (arrowhead), interspersed with mucous-secreting glands (hematoxylin and eosin stain; original magnification, ×200).

A xifo-umbilical median laparotomy was made; intraoperative findings confirmed the radiographic findings. In light of these findings, a subtotal gastric resection was performed, removing more than half of the stomach, followed by gastrojejunal anastomosis. Macroscopic examination revealed a lesion in the antrum extending 8cm along its longitudinal axis (Figure 4). The cut surface was grayish white and lobulated. On histology, the lesion showed features of a hamartomatous polyp with necrosis and ulceration of the mucosal surface. The lesion was composed of two proliferations, one composed of dilated pancreatic-type ducts surrounded by smooth muscle bands, interspersed with mucous secreting glands and the other containing Brunner’s glands (some of which were pseudoadenomatous) (Figure 5). This composition resembled a myoepithelial hamartoma.

The postoperative period was unremarkable and our patient’s general conditions were optimal at discharge two weeks after surgery. Our patient was followed-up at two months and six months after discharge with a complete physical examination and ultrasonography of his whole abdomen, and had no recurrence of symptoms.

## Discussion

A myoepithelial hamartoma of the stomach is a rare tumor. It is also referred to as myoglandular hamartoma, adenomyomatous hamartoma, gastric adenomyoma and heterotopia [5,6]. Lesions where pancreatic tissue is normally arranged are defined as an ectopic pancreas [7]; one third of patients may present with nests of pancreatic tissue dispersed in the smooth muscle bands. The exact pathogenesis and natural history of these lesions is not fully understood, possibly because very few cases have been reported in the literature. Generally, these lesions are either considered a form of hamartoma or a pancreatic heterotopia. Gal *et al.*[8] suggested that these lesions should be considered a form of hamartoma based on their observation of goblet cells, argentaffin cells and smooth muscle stroma. Alternatively, Takahashi and Fukusato [9] reported that these lesions demonstrated presence of cytokeratin 7 on immunohistochemical staining, which is distributed in the pancreatic duct epithelium, suggesting that the heterotopic pancreas theory may be more plausible. A properly structured follow-up study may explicate the natural history of myoepithelial hamartoma.

According to our research, 39 cases have been reported so far in the literature, not including ours [10]. In these cases, the patients’ age ranged from eight weeks to 81 years and in almost two thirds of them, the lesion was detected between the fourth and sixth decades of life [3]. The distinctive histological feature of this tumor was the presence of hypertrophic smooth muscle bands that surround the epithelial elements, arranged in various patterns including simple glandular, Brunner’s gland, and pancreatic acini. Ectopic pancreatic tissue nests were reported in one third of the cases [5,11] and were generally exocrine in nature, whereas the remainder had dilated pancreatic-like nests surrounded by smooth muscle bands, as in our case.

Lesions like these have been recorded in the gastric antrum (85%) and in the pylorus (15%) [4]. They usually present with nonspecific symptoms such as intermittent gastric pain, nausea and vomiting, and in some patients cause intermittent pyloric obstruction [4,5,11]. Among the cases reported in the literature, most were diagnosed by using conventional X-rays of the upper gastrointestinal tract using a barium meal follow-through; others were detected incidentally during surgery or autopsy [4,5,11]. The diagnosis had not been suspected prior to postoperative or postmortem histological findings, even in the cases where an esophagogastroduodenoscopy had been performed [4,5,11]. Because these tumors exhibit the form of a submucosal mass with central pancreatic heterotopia, an endoscopy does not permit a differential diagnosis between hamartoma and leiomyoma, melanoma, lymphoma, carcinoid tumor, Kaposi’s sarcoma or eosinophilic granuloma [11]. These hamartomas often may appear as a roundish- or oval-shaped mass in the submucosal tissue, simulating a lipoma, a neurofibroma or a polypoid formation.

Our case simulated a GIST, presenting on CT as an oval-shaped mass in his upper abdomen, compressing his duodenum. Endoscopy does not produce any useful biopsy for histology because most of these lesions, including the one in our case, are localized in the submucosal layer. Vandelli *et al.*[3] had attempted to treat an antral lesion unsuccessfully but had made a correct preoperative diagnosis of hamartoma as they had removed it piecemeal via endoscopy. However, this approach failed and confirmed the difficulty of preoperative diagnosis. These observations, along with the negative histology results, corroborate the recommendation made by Hedembro *et al.*[12] for surgery and resection in all cases of suspected gastric lesions to ensure a correct diagnosis. Radical surgery is justified in cases of a definite diagnosis of hamartoma as the lesion is very rare and may not be benign, and the risk of cancerization is still an open question [13]. In case of an adenomyoma of the periampullary region, surgical limited resection is recommended; however, pancreaticoduodenectomy is often performed as the lesion is frequently misdiagnosed as a carcinoma [14]. Whenever possible, perioperative frozen section diagnosis should be used to prevent unnecessary radical surgery.

## Conclusion

Gut masses warrant a thorough investigation and the presence of rare tumors should be excluded. Hamartomas can only be diagnosed by endoscopy and histological evaluation. The authoritative opinion is that a hamartoma should always be treated surgically, to avoid possible malignant degeneration or, as in rare instances, intestinal obstruction. The complete removal of the hamartoma can give complete remission of symptoms. Clinically, it is difficult to differentiate these lesions from a carcinoma, especially when they occur in the Vaterian system, often leading to a needless pancreaticoduodenectomy. To avoid unnecessary radical surgery, it is essential for clinicians to maintain current knowledge of the lesion and the most effective modality of treatment.

## Consent

Written informed consent was obtained from the patient for publication of this case report and any accompanying images. A copy of the written consent is available for review by the Editor-in-Chief of this journal.

## Competing interests

The authors declare that they have no competing interests.

## Authors’ contributions

JN and FNA analyzed and interpreted the patient’s clinical and radiological data and prepared the manuscript. JN performed the histopathological examination, prepared the pathology slides, and reviewed the manuscript. QA reviewed the manuscript and gave final input prior to publication. JN wrote the manuscript and prepared the radiology figures. All authors read and approved the final manuscript.
